# geneAttribution: trait agnostic identification of candidate genes associated with noncoding variation

**DOI:** 10.1093/bioinformatics/btw698

**Published:** 2016-11-29

**Authors:** Arthur Wuster, Diana Chang, Timothy W Behrens, Tushar R Bhangale

**Affiliations:** 1 Department of Human Genetics, Genentech Inc, South San Francisco, CA, USA; 2 Department of Bioinformatics and Computational Biology, Genentech Inc, South San Francisco, CA, USA

## Abstract

**Motivation:**

We have developed geneAttribution, an R package that assigns candidate causal gene(s) to a risk variant identified by a genetic association study such as a GWAS. The method combines user-supplied functional annotation such as expression quantitative trait loci (eQTL) or Hi-C genome conformation data and reports the most likely candidate genes. In the absence of annotation data, geneAttribution relies on the distances between the genes and the input variant.

**Availability and Implementation:**

The package is freely available from http://www.bioconductor.org/. A quick-start vignette is included with the package.

## 1 Introduction

The majority of variants identified by genetic association studies in humans are located in noncoding regions and likely act by affecting gene expression (Maurano *et al.*, 2012). In addition, variants typically contain multiple genes in their region of linkage disequilibrium, meaning that a naïve approach that simply designates the closest gene as the best candidate can be problematic.

**Fig. 1.**
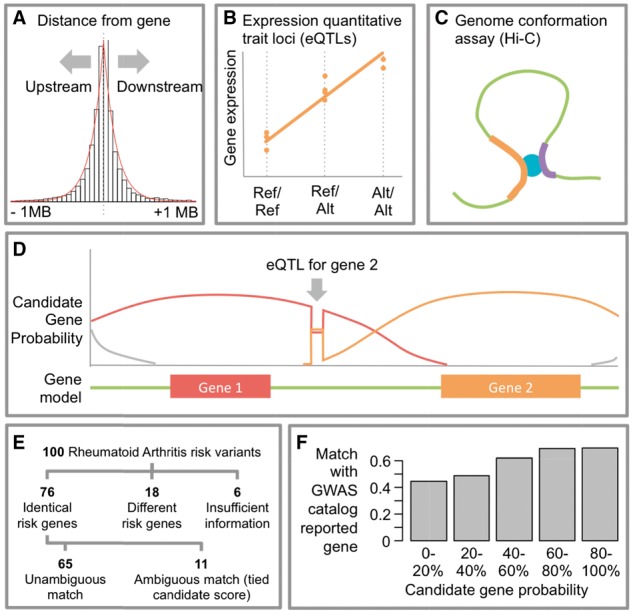
**A–C** Input data for geneAttribution. (A) Variant-gene distance, modeled as an exponential distribution (red line). (B) Expression quantitative trait loci (eQTLs), linking genotype to gene expression. (C) Hi-C genome conformation assays, linking gene promoters to distal regions. (**D**) Hypothetical example of candidate gene probabilities over a genomic region containing two genes. (**E**) Intersection between rheumatoid arthritis (RA) candidate genes predicted using a disease-specific score-based approach and our trait-agnostic approach. (**F**) Match of gene with highest causation probability and GWAS catalog reported gene for 21 512 associations

Given a genomic coordinate marking a variant identified by a genetic association study, geneAttribution aims to compute the relative probability for each of the genes in the vicinity of the variant. As a first step, the algorithm considers genes that are closer to the variant to be more likely candidate genes than those that are further away. To correctly calibrate the relationship between variant-gene distance and candidate gene probability, we used eQTLs from 36 tissues identified by the Genome-Tissue Expression Project (GTEx Consortium, 2015). We noted that the distance between the eQTLs and the genes they regulate can be approximated by an exponential distribution, with more eQTLs close to the gene than further away. An exponential distribution with λ = 7.61 × 10^−^^6^ fit the distribution of eQTLs both to the 5′ and the 3′ end of genes (Fig. 1A).

The geneAttribution algorithm can also take into account other user-supplied empirical data that links genomic regions to genes. Examples include expression quantitative trait loci (eQTLs) that link variants to the expression of specific genes (Fig. 1B) and Hi-C genome conformation data linking distal genomic regions to the promoters of specific genes (Fig. 1C). geneAttribution incorporates empirical data by first determining if any of the genomic regions specified in the empirical data overlap with the input variant. If there is no overlap, the empirical data are ignored. However, if there is an overlap, the likelihood of the associated gene is multiplied by a weight associated with the empirical data, which is also user-supplied. Finally, geneAttribution normalizes candidate gene likelihoods by dividing by the sum of the individual likelihoods (Fig. 1D).

More formally, we define the candidate probability as:

$$P\left(g_{j}|v\right)=\frac{P(v|g_{j})\cdot P(g_{j})}{\sum_{k=1}^{n}P(v|g_{k})\cdot {P(g}_{k})}$$

$$P\left(v|g_{j}\right)=\lambda \cdot e^{-\lambda \cdot d_{j}}$$

$$P\left(g_{j}\right)=\frac{1+\sum_{n=1}^{m}w_{j,n}\cdot Z_{j,n}}{\sum_{j=1}^{n}\left(1+\sum_{n=1}^{m}w_{j,n}\cdot Z_{j,n}\right)}$$

where *j* = 1, 2, …, *n*, with *n* being the number of genes within a given distance (default: 1MB) of the input variant *v*, *g_j_* is the *j*th gene, the parameter *λ* is set at a default of 7.61 × 10^−^^6^, *d_j_* is the distance between the variant *v* and the transcription start or end site (whichever is closer to *v*) of gene *g_j_*, *w_j,n_* is the user-assigned weight for gene *g_j_* in empirical dataset *n*, with *n *=* *1, 2, …, *m*, with *m* being the number of empirical datasets and *Z_j,n_* is an indicator function of whether any of the genomic locations associated with gene *g_j_* in dataset *n* overlap variant *v*.

## 2 Method assessment

To assess the performance of geneAttribution, we ran the program with default parameters and two types of empirical data. The first type was based on eQTLs from GTEx. Instead of only using the eQTL variants themselves, we defined a region around each eQTL in which the associated gene is given an elevated likelihood of being a candidate gene. We defined these regions based on flanking recombination hotspots as discovered by the HapMap project (International HapMap Consortium, 2005). We used the recombination sites with a recombination rate of more than 0.1 cM/Mb to define haplotype blocks. Only haplotype blocks that were smaller than 10 000 bases, representing 98.6% of all haplotype blocks, were used. To make our approach more tissue agnostic, only eQTLs present in more than one of the 36 GTEx tissues were used. The second empirical data type was based on high-resolution Capture Hi-C data (Mifsud *et al.*, 2015; Fig. 1C). Hi-C is a genome conformation assay that examines long-range genomic interactions of gene promoters. We assigned a weight of 5 to the eQTL data and a weight of 2 to the Hi-C data, reflecting our assumption that the eQTL data is more relevant than the Hi-C data when assigning candidate genes.

We then compared geneAttribution to a previously published approach that made extensive use of a variety of trait-specific data types to identify candidate genes at 100 rheumatoid arthritis risk loci (Okada *et al.*, 2013). To make results obtained by Okada *et al.* and those by geneAttribution comparable, we only included genes also considered by Okada *et al.* (3.8 genes per locus on average). Even though geneAttribution is trait agnostic, it identified the same candidate gene for 76 of the 94 loci (81%) for which such a determination was possible, and a different gene for 18/94 (19%) (Fig. 1E). For the remaining 6 loci, all candidate genes examined by Okada *et al.* were too far from the locus when using default parameters, making computation of candidate gene probabilities impossible (Fig. 1E). A naïve alternative approach designating the closest gene as the candidate gene only identified the same candidate for 71 of the 94 loci (76%). This indicates that incorporating empirical data improved accuracy.

We also applied geneAttribution to 21 512 associations in the NCBI/EBI GWAS catalog (Welter *et al.*, 2014) and found that the resulting candidate gene probabilities correlate with the probability that a gene has been annotated by the submitters to the catalog as a reported causative gene (Fig. 1F). Even though most of the reported causative genes have not been experimentally validated, this result suggests that the candidate gene probabilities that geneAttribution returns are well calibrated.

## 3 Conclusions

While we limited ourselves to Hi-C and eQTL data, users can easily also include any other empirical data linking genomic regions to candidate genes when using geneAttribution. These data types could either be generic or tissue or condition specific. An example of the latter would be ChIP-sequencing data for transcription factors that are relevant to the trait being studied. 

In summary, geneAttribution is a tool for the identification of likely candidate genes for variants of interest. While it is not a substitute for in-depth experimental validation, it can quickly suggest candidate genes without the need to first collect trait or disease specific data.
